# Supplementary material for: A neural signature for the subjective experience of threat anticipation under uncertainty
Source: Nat Commun. 2024 Feb 20;15:1544. doi: 10.1038/s41467-024-45433-6 (PMC10879105; doi:10.1038/s41467-024-45433-6)
Supplement: Supplementary file 3 — Reporting Summary [file 41467_2024_45433_MOESM3_ESM.pdf]

nature portfolio

Corresponding author(s): Benjamin Becker

Last updated by author(s): Dec 26, 2023

Reporting Summary

Nature Portfolio wishes to improve the reproducibility of the work that we publish. This form provides structure for consistency and transparency in reporting. For further information on Nature Portfolio policies, see our Editorial Policies and the Editorial Policy Checklist.

Statistics

For all statistical analyses, confirm that the following items are present in the figure legend, table legend, main text, or Methods section.

n/a	Confirmed	
[ ]	[x]	The exact sample size (n) for each experimental group/condition, given as a discrete number and unit of measurement
[ ]	[x]	A statement on whether measurements were taken from distinct samples or whether the same sample was measured repeatedly
[ ]	[x]	The statistical test(s) used AND whether they are one- or two-sided<br>*Only common tests should be described solely by name; describe more complex techniques in the Methods section.*
[ ]	[x]	A description of all covariates tested
[ ]	[x]	A description of any assumptions or corrections, such as tests of normality and adjustment for multiple comparisons
[ ]	[x]	A full description of the statistical parameters including central tendency (e.g. means) or other basic estimates (e.g. regression coefficient) AND variation (e.g. standard deviation) or associated estimates of uncertainty (e.g. confidence intervals)
[ ]	[x]	For null hypothesis testing, the test statistic (e.g. F, t, r) with confidence intervals, effect sizes, degrees of freedom and P value noted<br>*Give P values as exact values whenever suitable.*
[x]	[ ]	For Bayesian analysis, information on the choice of priors and Markov chain Monte Carlo settings
[x]	[ ]	For hierarchical and complex designs, identification of the appropriate level for tests and full reporting of outcomes
[ ]	[x]	Estimates of effect sizes (e.g. Cohen's d, Pearson's r), indicating how they were calculated

Our web collection on statistics for biologists contains articles on many of the points above.

Software and code

Policy information about availability of computer code

Data collection	MRI data was collected using a GE Discovery MR750 3.0T scanner (General Electric Medical System, Milwaukee, WI, USA). Skin conductance was measured during MRI scanning using a MRI-compatible Biopac system (MP-150, Biopac Systems Inc., Goleta, CA). Electric stimulation was delivered via a Biopac STM100C. Behavioral data was collected using E-Prime 2.0 (Psychology Software Tools, Sharpsburg, PA). Self-reported personality questionnaires were filled out online via SurveyCoder 3.0 (https:// www. surveycoder. com/).
Data analysis	fMRI preprocessing and data analysis were performed using Statistical Parametric Mapping software (SPM12; Wellcome Department of Cognitive Neurology, London, UK) in Matlab R2020b environment and CanlabCore Tools (https:// github.com/canlab/CanlabCore) in addition to custom Matlab code (https://github.com/lucyliu666/Anxiety_decoder or https://osf.io/a8gcb/). Skin conductance data was preprocessed using Biopac Acqknowledge 4.2.0 software and custom Matlab code (https://github.com/lucyliu666/Anxiety_decoder or https://osf.io/a8gcb/). Behavioral data was analyzed via R (version 4.3.1, code see https://osf.io/a8gcb/).

For manuscripts utilizing custom algorithms or software that are central to the research but not yet described in published literature, software must be made available to editors and reviewers. We strongly encourage code deposition in a community repository (e.g. GitHub). See the Nature Portfolio guidelines for submitting code & software for further information.

Data

Policy information about availability of data

All manuscripts must include a data availability statement. This statement should provide the following information, where applicable:

- Accession codes, unique identifiers, or web links for publicly available datasets
- A description of any restrictions on data availability
- For clinical datasets or third party data, please ensure that the statement adheres to our policy

The data that has been used to develop and evaluate the SUITAS are available on the Open Science Framework (https://osf.io/a8gcb/). The meta-analytic map comparing uncertain threat versus safe anticipation in healthy individuals is available at https://neurovault.org/images/384665/.

Research involving human participants, their data, or biological material

Policy information about studies with human participants or human data. See also policy information about sex, gender (identity/presentation), and sexual orientation and race, ethnicity and racism.

Reporting on sex and gender	The findings apply to all genders. No gender-based analysis was performed because this work did not have a prior hypothesis related to gender difference. Gender was not considered in the study design either. The information on gender was collected based on self-reported measures and was included in each study.
Reporting on race, ethnicity, or other socially relevant groupings	All participants in the original datasets were self-reported Asian (Chinese) university students based on the "Ethnicity" reporting on the written informed consent provided by the researchers, which was indicated in the Methods section of the manuscript. There were no classification terms for this item to choose and the participants were asked to provide the information by writing it down. The Ethnicity information for all datasets was provided in Supplementary Table 1.
Population characteristics	-Study 1: 44 healthy participants (23 females, age = 22.07 ± 2.50[mean ± SD]).<br>-Study 2: 30 healthy participants (14 females, age = 22.47 ± 2.76[mean ± SD]).<br>-Study 3: 50 healthy participants (27 females, age = 20.08 ± 2.22[mean ± SD]).<br>-Study 4: This dataset was from our previous study. 59 healthy participants (all males, age = 20.68 ± 1.74[mean ± SD]).<br>-Study 5: This dataset was obtained from https://osf.io/68ywz/, a publicly available dataset. 68 healthy participants (45 females, age = 29.64 ± 15.89[mean ± SD]).<br>-Study 6: This dataset was obtained from https://figshare.com/articles/dataset/bmrk3_6levels_pain_dataset_mat/6933119, a publicly available dataset. 33 healthy participants (22 females, age = 27.9 ± 9.0[mean ± SD]).<br>-Study 7: This dataset was taken from an ongoing study from College Student Cohort of Zhangjiang International Brain Biobank (https://zib.fudan.edu.cn). 100 healthy participants (63 females, age = 18.68 ± 0.87[mean ± SD]).<br>-Study 8: This dataset was from the same sample in Study 3, in which data from 2 participants were missing. 48 healthy participants (25 females, age = 20.10 ± 2.20[mean ± SD]).<br>-Study 9: This dataset was a subsample of Study 2 and 3. 65 healthy participants (33 females, age = 20.89 ± 2.17[mean ± SD]).<br>-Study 10: This dataset was obtained from https:// figshare.com/articles/dataset/Subjective_fear_dataset/1327110, a publicly available dataset. 67 healthy participants (34 females, age = 21.5 ± 2.1[mean ± SD]).<br>-Study 11: This dataset was obtained from https://neurovault.org/collections/1964/, a publicly available dataset. 121 healthy participants.
Recruitment	Participants for Studies 1-3 were recruited via online advertisement on Tencent QQ and all participants were Chinese university students in UESTC. Participants were healthy, with normal and corrected to normal vision, and free of current or history of neurological and major physical disease, mood or anxiety disorders, psychotropic medications, substance abuse and any prior participation in experiments with electric stimulation. All participants provided written informed consent and were remunerated 120 RMB for their participation. Subjects recruited for this work from the UESTC may not be representative of the entire population of healthy university students in all universities and healthy adults in other communities, age groups and races/ethnicities.
Ethics oversight	All studies were approved by the local ethics committee at the University of Electronic Science and Technology of China.

Note that full information on the approval of the study protocol must also be provided in the manuscript.

Field-specific reporting

Please select the one below that is the best fit for your research. If you are not sure, read the appropriate sections before making your selection.

[x] Life sciences [ ] Behavioural & social sciences [ ] Ecological, evolutionary & environmental sciences

For a reference copy of the document with all sections, see nature.com/documents/nr-reporting-summary-flat.pdf

Life sciences study design

All studies must disclose on these points even when the disclosure is negative.

Sample size	The sample size of Study 1 (n = 44) was determined based on previous publications that had a similar goal (e.g., Ceko et al., 2022, Nature

	Neuroscience; Lee et al., 2021, Nature Medicine). Moreover, we included validation and independent test datasets (Study 2, n = 30; Study 3, n = 50) to more comprehensively test the model sensitivity and generalizability, with sample sizes compared to previous studies (Kragel et al., 2023, Nature Human Behaviour; Zhou et al., 2021, Nature Communications) and even larger. No formal statistical procedures were used to determine sample size for Studies 4-11 because these studies analyzed existing datasets.
Data exclusions	No data were excluded from the analyses.
Replication	Model performance was verified in 4 validation and generalization cohorts (Studies 2-5) that support replication. The specificity of the model was assessed in 6 independent datasets (Studies 6-11).
Randomization	All subjects completed four fMRI runs of the UVTA task. We pseudo-randomized the order of experimental conditions within each run and counterbalanced the run orders across participants. In all experiments no between-subjects experimental groups were created.
Blinding	No blinding was performed in this work.

Reporting for specific materials, systems and methods

We require information from authors about some types of materials, experimental systems and methods used in many studies. Here, indicate whether each material, system or method listed is relevant to your study. If you are not sure if a list item applies to your research, read the appropriate section before selecting a response.

Materials & experimental systems

n/a	Involved in the study	
☒	☐	Antibodies
☒	☐	Eukaryotic cell lines
☒	☐	Palaeontology and archaeology
☒	☐	Animals and other organisms
☒	☐	Clinical data
☒	☐	Dual use research of concern
☒	☐	Plants

Methods

n/a	Involved in the study	
☒	☐	ChIP-seq
☒	☐	Flow cytometry
☐	☒	MRI-based neuroimaging

Plants

Seed stocks	*Report on the source of all seed stocks or other plant material used. If applicable, state the seed stock centre and catalogue number. If plant specimens were collected from the field, describe the collection location, date and sampling procedures.*
Novel plant genotypes	*Describe the methods by which all novel plant genotypes were produced. This includes those generated by transgenic approaches, gene editing, chemical/radiation-based mutagenesis and hybridization. For transgenic lines, describe the transformation method, the number of independent lines analyzed and the generation upon which experiments were performed. For gene-edited lines, describe the editor used, the endogenous sequence targeted for editing, the targeting guide RNA sequence (if applicable) and how the editor was applied.*
Authentication	*Describe any authentication procedures for each seed stock used or novel genotype generated. Describe any experiments used to assess the effect of a mutation and, where applicable, how potential secondary effects (e.g. second site T-DNA insertions, mosiacism, off-target gene editing) were examined.*

Magnetic resonance imaging

Experimental design

Design type	Event-related design
Design specifications	Four cue types signaling four uncertainty levels for a total of 96 randomized trials were distributed over four fMRI runs (24 trials per run). Each trial began with a 4-6 s fixation-cross followed by a fixed 8 s (blue) or 6-10 s (avg 8 s) anticipatory cue (purple, red and white) period. When the anticipatory cues disappeared, an outcome (either 2-3 shocks or no shocks depending on the color of the cue) was quickly delivered to the participants and a black screen was presented for 750 ms. After a 2-4 fixation jitter screen, the participants reported how much anxiety they experienced during the anticipatory cue presentation phase on a Likert scale of 1-5 (1 = no anxiety to 5 = extreme anxiety) within 4 s.
Behavioral performance measures	Behavioral data (rating of anxious arousal during the anticipation period) was collected on a Likert scale of ranging from 1-5. Mean within- and across- participant ratings ± SE for each condition are presented in Figure 1 and in Supplementary Figure 1.

Acquisition

Imaging type(s)	Functional and structural
Field strength	3 Tesla
Sequence & imaging parameters	Gradient echo-planar imaging sequences (40 slices; repetition time (TR) = 2000 ms; echo time (TE) = 30 ms; slice thickness = 3.8 mm; spacing = 0.6 mm; field of view (FOV) = 200 × 200 mm; flip angle = 90°; matrix size = 64 × 64; voxel size = 3.125 × 3.125 × 3.8mm). High-resolution whole-brain T1-weighted images were additionally acquired to improve spatial normalization (3D spoiled gradient echo pulse sequence; 154 slices; TR = 6 ms; , TE = 3 ms; slice thickness = 1mm, FOV = 256 × 256 mm, acquisition matrix = 256 × 256, flip angle = 8°, voxel size = 1 × 1 × 1 mm).
Area of acquisition	Whole brain

Diffusion MRI ☐ Used ☒ Not used

Preprocessing

Preprocessing software	The fMRI data were preprocessed using Statistical Parametric Mapping (SPM12, https://www.fil.ion.ucl.ac.uk/spm/software/spm12/) and CanlabCore (https://github.com/canlab/CanlabCore).
Normalization	T1 image was normalized to MNI space using affine and non linear transformations as implemented in SPM12.
Normalization template	SPM12's MNI normalized template
Noise and artifact removal	The first five volumes of each run were discarded to allow for magnetic field equilibration. Prior to preprocessing, image intensity outliers were identified using CanlabCore tools (https://github.com/canlab/CanlabCore). Each time-point identified as outliers was included in the first-level model as a separate nuisance covariate. 24 head motion parameters (6 realignment parameters demeaned, their derivatives and the squares of these 12 regressors) and indicator vectors specifying “spikes” by framewise displacement (FD) that had deviations larger than 0.50 mm were also included as nuisance regressors.
Volume censoring	No censoring, instead inclusion of motion spikes as regressors in the first-level model

Statistical modeling & inference

Model type and settings	Brain models predicting self-reported anxiety ratings (continuous outcome) during anticipation of uncertain electric shocks.
Effect(s) tested	To provide an interpretable effect size metric, we calculated Pearson correlations between the predicted ratings and the actual ratings across participants for Studies 1-3, and the statistical inference was determined using a permutation test with 5,000 random shuffles for Study 2 and 3. To test the predictive performance of the shock uncertainty-induced threat anticipation signature (SUITAS), we calculated the explained variance score (EVS) to indicate the overall prediction error. To determine which brain areas made reliable contributions to the prediction and to facilitate interpretation and display, we constructed 5,000 bootstrap samples (with replacement) consisting of paired brain and outcome data and repeated SVR on each bootstrap sample. The bootstrap distribution was then converted into Z values and the model weight map was thresholded based on the corresponding P values (two-tailed, uncorrected P < 0.001).

Specify type of analysis: ☐ Whole brain ☐ ROI-based ☒ Both

Anatomical location(s)	The anatomical regions for ROI-based analysis and the corresponding atlases used to select ROIs are detailed in Supplementary Table 3.
Statistic type for inference (See Eklund et al. 2016)	Voxel-wise
Correction	FDR correction

Models & analysis

n/a	Involved in the study
☒	☐ Functional and/or effective connectivity
☒	☐ Graph analysis
☐	☒ Multivariate modeling or predictive analysis

Multivariate modeling and predictive analysis	Support vector regression was conducted using Spider toolbox (http:// people.kyb.tuebingen.mpg.de/spider) in MATLAB (R2020b). Predictors (X) constituted 198 whole-brain activation maps associated with participants (44) x self-reported anxiety level (3~5), aggregated into an images x voxels matrix (stacked across participants), and split into training and test sets using 10×10-fold cross-validation. The activation maps were derived via univariate GLM analysis in which we modeled anticipation periods (of no shock trials) against implicit baseline and averaged across trials of each rating level to obtain a maximum of 5 contrast images per participant. To evaluate the model performance and minimize overfitting, we used 10 × 10-fold cross-

validation within the training dataset in Study 1 (n = 44) and applied the brain model (i.e., SUITAS) to new individuals in Study 2 (n = 30) and Study 3 (n = 50). Signature responses were calculated using the dot product of each pattern weight map with the univariate GLM-derived vectorized activation map for each participant for each rating level. The population-level weight map (SUITAS) predictive of subjective anxiety levels was generated by taking 5,000 samples with replacement from the training dataset (N = 44), repeated the prediction process with each bootstrap sample, and calculated Z scores and two-tailed uncorrected P values with the mean and standard deviation of the sampling distribution. The within-individual pattern weight map was obtained from one-sample t test of weight maps for all participants using their single-trial beta images (maximum of 44 contrast images, 10×10-fold cross-validated) and was corrected at FDR q < 0.05.
